# Head & Neck Oncology: purpose, scope and goals-charting the future

**DOI:** 10.1186/1758-3284-1-1

**Published:** 2009-01-12

**Authors:** Adel K El-Naggar, Colin Hopper, Tahwinder Upile, Waseem Jerjes

**Affiliations:** 1 Head and Neck Pathology, Division of Pathology, UT. M.D. Anderson Cancer Center, Texas, USA; 2 Head & Neck Centre, University College London Hospitals, London, UK; 3 Unit of Oral & Maxillofacial Surgery, Division of Maxillofacial, Diagnostic, Medical and Surgical Sciences, UCL Eastman Dental Institute, London, UK; 4 Department of Surgery, University College London Medical School, London, UK; 5 Department of Head & Neck Surgery, the Professorial Unit, the Royal National Throat, Nose and Ear Hospital, London, UK

## Abstract

For many years now there has been a growing frustration with the statistics of head and neck cancer. Despite the many advances in diagnosis and therapy, there has been little change in the prognosis for most cancers of the head and neck in the last 50 years, so what is the point of yet another journal? Well, it is not all bad news.

## Editorial

Standardisation of protocols and therapies have at least ironed out some inconsistencies in the care delivered and much of the work on quality of life issues has better informed the process of treatment planning. Patients are no longer willing to accept a treatment that has been recommended by an individual with intrinsic prejudices. They need the facts about the impact of therapy on their function and appearance as well as a realistic assessment of their prognosis. Too often in the past, patients have been offered extensive surgery as the only chance of a cure when the survival statistics suggest these chances are very slim.

These factors are of course applicable to many parts of the world, but in underprivileged societies, the problems are even more complex if there is no infrastructural support to comprehensively treat those who suffer from cancer of the head and neck.

Starting this new journal, *Head & Neck Oncology*, is an undertaking that requires a conceptually clear vision of its goals and distinctiveness from existing publications and an intellectual involvement of Editorial Board members. This is especially true in today's widely disseminated media, which offer a broad menu of similar and often overlapping topics. Head and neck oncology publications have traditionally been surgically oriented due to the dominance of the practice for these neoplasms. In the past two decades, however, two major developments have emerged that have fundamentally transformed the head and neck cancer oncology field. One is the introduction of adjuvant chemotherapy in advanced laryngeal cancer, and the second is the successful use of concurrent chemo and radiotherapy treatment in operable late stage patients. As a result, multimodality concepts and demand for surrogate biomarkers have emerged as a dominant topic and have led to an intense research in several fields. To this end, the journal will provide a platform for articles from diverse subjects linking many head and neck areas for the readers and contributing authors. To accomplish this, an Editorial Board that represents a wide range of expertise in different fields has been assembled and charged with the responsibility to provide an efficient, balanced review process, all with high standards for presentation to our readers.

The realisation of the importance of multidisciplinary team working has also had an impact on quality of life issues. However, there still persists a divide between surgeons and radiation oncologists, not to mention medical oncologists and allied health professionals. We intend to bridge this gap by publishing, commissioning and soliciting articles from all the personnel involved in the care of head and neck cancer patients.

While *Head & Neck Oncology *will serve as a vehicle for advances in squamous mucosal cancer, it will also present topics from underserved and so-call "orphan tumors" of the head and neck, including those of salivary gland, soft tissue and skeletal neoplasms, endocrine, neuroendocrine neoplasms, and the others that make head and neck oncology arguably the most challenging of all oncology. Another important focus of the journal will be the fields of diagnostic and therapeutic biomarkers as a guide in treatment of patients with surgically unresected, recurrent and metastatic salivary gland, thyroid and sinonasal tumors. Similarly, studies on the best practices and standardization of translational, biological, clinical practices and quality assurance programs will be solicited and encouraged. This diversity will serve readers with information to minimize the variability of the diagnostic and clinical staging within and across countries and continents. It will also encourage the development of common basic elements for clinical, pathological, and radiological assessment of patients and the development of risk assessment modules that advance and guide therapy. Banking, standardization of harvesting, storing and transportation of head and neck tumors and patient materials among countries and continents is an emerging field of interest to the journal. Studies in this field addressing these activities are important for collaborative research multi-centre clinical trials and correlative science. Likewise, rehabilitation and functional outcome of head and neck cancer patients are nascent but critical topics for the readers and interested contributors. The time has come for the development and implementations of semi-quantitative and objective measures to assess quality of life of these patients, and the topic will be welcomed by the journal.

### Our interests although not limited to, will include

#### Early diagnosis

Screening of high-risk groups with simple, semi automated techniques such as elastic scattering spectroscopy has the potential to deliver a rapid accurate diagnostic service. In the future, this might be offered outside the conventional settings of a medical or dental practice. We see this as being an exciting area of research that has shown early promise although refinement will be necessary to make the technology widely applicable.

#### Therapeutic advances

While some advances develop by a gradual process of evolution and better tumour targeting; for example intensity modulation and conformal radiotherapy – others happen very rapidly by the application of new approaches. Photodynamic therapy, for example, while first described over a century ago is now licensed in the management of advanced head and neck cancers in Europe and is an effective addition to our therapeutic arsenal. New chemotherapy approaches with epithelial growth factor receptor blockers such as Cetuximab have shown benefit in combination with radiotherapy and there are now many candidate compounds that are under investigation. We all await the day when some "magic bullet" will leave us seeking alternative employment, but to date, there have only been tantalising glimpses of what might be. We will welcome 'tips and tricks' contributions that colleagues may have developed which help them optimise care be it surgery, radiotherapy, oncology...etc.

There is also a reluctance to accept new treatments in mainstream clinical practice. Much of this conservatism is a result of a paucity of level 1 evidence for almost any treatment of head and neck cancer, especially surgical studies. Funding for appropriately designed studies needs to be secured to better dictate treatment decisions. Traditionally, this has proved difficult because head and neck cancer typically (although not exclusively) affects the older populations who smoke and drink alcohol. This group are not the most vocal or articulate regarding their condition and lack the emotional undertones of paediatric malignancy, colon, breast and prostate cancer, which are non-selective in terms of social class of the population at risk. The formation of collaborative groups with sufficient numbers to complete studies in a timely fashion has economies of scale, but more importantly show a commitment to serious research that should help funding.

#### Outcome measurements

Outcome is now a major factor dictating treatment decisions and funding allocations. For patients suffering from severe symptoms and/or side effects, quality of life outcomes is every bit as important as survival rates. For example, more targeted delivery of radiotherapy with maximal shielding of salivary gland tissue becomes possible when patients with radiation-induced xerostomia communicate their feelings about which balance of survival rates and being able to live without a dry mouth the treatment should aim for. It is also an area in which speech and language therapists and dieticians are able to quantify the adverse effects of treatment. We will aim to avoid publishing bias and support both positive and refuting research.

#### Treatment failure and Palliative Care

It is an unfortunate fact of life in head and neck cancer that treatments may fail, and patients may require palliative care. However, clinicians have a lifelong commitment to their patients and it is unreasonable to disengage and merely refer patients to a hospice. The role of the clinical nurse specialist is key throughout the patient journey, but rarely more important than at the stage of disease recurrence. Patients may be offered any number of palliative therapies and they will need help in deciding on exactly what treatment is reasonable. This should be seamlessly integrated with palliative care input and patients should expect sufficient input to support their needs. Fear of death is often associated with fear of the unknown or concerns about pain control. Thanks to the pioneering work of Dame Cicely Saunders and others, symptom relief has developed to the stage where all palliative patients are made comfortable and allowed to die with dignity.

It is our aim with this journal to encourage the interface between all clinicians involved in management of head and neck cancer with a truly multidisciplinary approach. The next 10 years should be a fertile period in our very specialised area of head and neck oncology.

Finally, there are many challenges that are ahead of us, among which are to ensure high quality articles and original studies in a competitive field, keeping pace with new developments, discoveries and providing a timely and fair review process. We hope that you will help support *Head & Neck Oncology *and the future of this field.

